# Tripchlorolide attenuates β-amyloid generation by inducing NEP activity in N2a/APP695 cells

**DOI:** 10.1515/tnsci-2020-0178

**Published:** 2021-07-20

**Authors:** Yuqi Zeng, Yongkun Li, Hui Shen, Nan Lin, Jian Zhang

**Affiliations:** Department of Neurology, Institute of Clinical Neurology, Fujian Medical University Union Hospital, 29 Xinquan Road, Fuzhou, Fujian, 350001, China; Key Laboratory of Brain Aging and Neurodegenerative Disease, Institute of Clinical Neurology, Fujian Key Laboratory of Molecular Neurology, Fujian Medical University, 88 Jiaotong Road, Fuzhou, Fujian, 350001, China; Department of Neurology, Xiamen Key Laboratory of Brain Center, The First Affiliated Hospital of Xiamen University, Xiamen, Fujian, 361002, China; Department of Geriatrics, Fujian Institute of Geriatrics, Fujian Medical University Union Hospital, 29 Xinquan Road, Fuzhou, Fujian, 350001, China; The School of Clinical Medicine, Fujian Medical University, 88 Jiaotong Road, Fuzhou, Fujian, 350001, China

**Keywords:** tripchlorolide, neprilysin, amyloid-β protein, N2a/APP695 cells

## Abstract

**Background and purpose:**

Alzheimer’s disease (AD) is a neurodegeneration disease. The previous work from our research group demonstrated the neuroprotective effects of tripchlorolide (T4) in AD animal models.

**Materials and methods:**

Neprilysin (NEP) is known as an important physiological amyloid-β protein (Aβ) peptide-degrading enzyme in the brain due to its apparent rate-limiting function. In this study, we explored the effect of NEP on AD model N2a/APP695 cells. Western blots and enzyme-linked immunosorbent assays were performed to assess the expression of proteins, while quantitative real-time polymerase chain reaction assays were used to evaluate RNA levels. Cell vitality was detected by the MTT assay, and reactive oxygen species (ROS) levels were assessed using a ROS activity assay kit.

**Results:**

We discovered that T4 was able to enhance the enzyme activity of NEP. T4 administration decreased the protein levels of the soluble amyloid precursor protein. In further experiments, we found that by using thiorphan the secretion of Aβ, oxidative stress, nitrosative stress, and inflammatory factors, which were suppressed by T4, were reversed. Due to its ability to attenuate Aβ generation and to protect neurons against the neurotoxicity of Aβ, T4 may be a potential therapy in the regulation of Aβ-related pathology in AD by affecting NEP activity.

**Conclusion:**

Tripchlorolide attenuates Aβ generation by inducing NEP activity in N2a/APP695 cells.

## 1 Introduction

Alzheimer’s disease (AD) is a common neurodegenerative disease as well as the most frequent cause of dementia, affecting over 30 million people around the world [1]. The incidence of AD is related to aging, and therefore, when life expectancy increases so is the morbidity of AD [2]. Though enormous efforts have been made to advance AD treatment options, no helpful disease therapeutic is yet available, partially due to our limited knowledge of the pathogenesis of AD [3].

Senile plaques are considered to be the pathological marker of AD, consisting of amyloid-β protein (Aβ), which can lead to secondary pathological changes, including hyperphosphorylation of tau, oxidative stress, and neuroinflammation, as well as neurite degeneration, eventually resulting in dementia [4]. The soluble oligomeric form of Aβ peptide has been proven to be toxic to neuronal synapses, and the accumulation of Aβ peptide plays a vital role in the progression of AD [5]. In addition, studies have found that the accumulation of Aβ in the brain can lead to neuroinflammation, which may cause a reduction in synapses and the loss of neuronal function [6]. As a result, preventing Aβ peptide accumulation in the brain represents a potential way to prevent memory loss and neuronal death in AD.

Some enzymes, containing angiotensin-converting enzyme, endothelin-converting enzyme, matrix metalloproteinases, insulin-degrading enzyme, plasmin, and neprilysin (NEP), can degrade Aβ peptide [7]. Among them, NEP is particularly known as a key physiological Aβ peptide-degrading enzyme in the brain [8]. Previous research has reported that NEP levels are decreased in areas that are vulnerable to the accumulation of Aβ peptides in the brains of AD patients [9]. Therefore, upregulation of the activity and level of NEP in the brains of AD patients may attenuate Aβ peptide accumulation, protect neurons against Aβ toxicity, and help inhibit cognitive deficits as well as Aβ-related synaptic loss [10].

Tripchlorolide (T4), an extract of a traditional Chinese herbal *Tripterygium wilfordii* Hook F., is a diterpene triepoxide [11]. T4 has potent anti-inflammatory as well as immunosuppressive effects and results in minor toxicity. It is able to cross the blood–brain barrier due to its lipophilicity and small molecular size [12]. It has been proven to improve age-associated cognitive deficits, synapse-related receptor dysfunction, and impaired hippocampal long-term potentiation [13]. A recent study has revealed that T4 increases the expression of hippocampal neuroligin-1 in AD mice by epigenetic mechanisms, offering a novel explanation for the mechanisms underlying the protective effect of T4 on synapses [14]. Furthermore, our previous experiments have found that T4 can improve cognitive deficits by decreasing Aβ and increasing levels of synapse-related proteins in an AD model [15]. However, whether T4-induced Aβ reduction is associated with NEP expression remains to be elucidated.

In this study, we explored the effect of T4 on NEP expression and its role on Aβ degradation and secretion in mouse neuro-2a (N2a) cells stably expressing human APP695, which is always used as a model of Aβ generation *in vitro*. Our results provided a basis for clarifying the role of T4 in the treatment of AD and finding new AD therapies. This study’s results may help advance the efficient use of T4 in the clinical treatment of AD.

## 2 Materials and methods

### 2.1 Cell culture

N2a/APP695 cells, derived from a mutated APP-overexpressing neuronal cell line, were used to mimic APP metabolism and Aβ generation *in vitro*. N2a cells stably expressing human APP695 were gifts kindly provided by Professor Qinwen Wang (Ningbo University, Zhejiang, China). Cells were cultured in Dulbecco’s modified Eagle’s medium (DMEM)/Opti-MEM (1:1; containing 5% fetal bovine serum (FBS), 200 μg/mL G418 (geneticin), 100 mg/mL streptomycin, 100 U/mL penicillin; Gibco BRL, Grand Island, NY) and kept at 37°C in 5% CO_2_. N2a cells were passaged every 3 days when growing up to 80% confluence. For assay experiments, cells were withdrawn from mitogen stimulation for 1 day prior to the assays and then treated with 5 nM or 10 nM T4 (2300158, Seebio Biotechnology, Shanghai, China) for 24 h. Some cells were exposed to thiorphan (500 nM; Cayman Chemical, Ann Arbor, MI) for 1 h prior to T4 administration (10 nM) for 24 h.


**Ethical approval:** The conducted research did not require an ethical board approval because it did not contain human or animal trials.

### 2.2 MTT assay

The cell survival rate was assessed by an MTT assay kit (11465007001, Roche Diagnostics, Mannheim, Germany) following the manufacturer’s instructions. Following treatment with indicated regents, the culture medium was replaced with the medium containing 0.5 mg/mL MTT. Thereafter, the medium was replaced with a solubilization solution for further incubation overnight. The optical density (OD) value was assessed at 570 nm by an automated microtiter plate reader.

### 2.3 Quantitative real-time polymerase chain reaction (qRT-PCR)

Total RNA was extracted from cells with the Trizol reagent according to instructions. Next, 500 ng of total RNA was reverse-transcribed into cDNA using a ThermoScript RT system (Thermo Fisher Scientific, Waltham, MA). PCR amplification was carried out through an initial denaturing step at 94°C for 5 min, followed by 30 cycles at 94°C for 45 s, at 58°C for 45 s and at 72°C for 45 s, and a further extension at 72°C for 10 min. The PCR products were electrophoresed through a 1% agarose gel and visualized with ethidium bromide staining. The relative expressions of mRNA were normalized with glyceraldehyde-3-phosphate dehydrogenase (GAPDH).

### 2.4 Western blot analysis

Cultured cells or tissues were lysed with sodium dodecyl sulfate (SDS) sample buffer and boiled for 10 min. The protein content was normalized using protein assay kits (Bio-Rad, Hercules, CA). Equal amounts of total protein were separated by SDS polyacrylamide gel electrophoresis, electrophoretic transferred to a polyvinylidene-fluoride membrane, and immunoblotted with primary antibodies (Neprilysin/CD10 antibody, NBP2-15771, Novus Biologicals, Littleton, CO; GAPDH polyclonal antibody, 2172, 10494-1-AP, Proteintech, Hubei, China) overnight. After washing three times using tris-buffered saline with Tween (TBST), the membranes were incubated with horseradish peroxidase-conjugated secondary antibody (Santa Cruz Biotechnology, Santa Cruz, CA) for 2 h. After washing thrice with TBST, the secondary antibodies were visualized using the LumiGLO Reagent (Cell Signaling Technology, Danvers, MA).

### 2.5 Aβ and inflammatory factor assay

The concentrations of Aβ1-42 (CSB-E10787m, Cusabio, Hubei, China), Aβ1-40 (CSB-E08300m,Cusabio), IL-1β (SEKM-0002, Solarbio, Beijing, China), and TNF-α (SEKM-0034, Solarbio) were assessed by enzyme-linked immunosorbent (ELISA) assay kits. In brief, cells were seeded onto 96-well plates and treated under different conditions. Thereafter, 100 μL of supernatants was harvested to perform ELISA assays. Nitric oxide levels were detected according to total nitrate/nitrite ratios in the supernatants following the manufacturer’s instructions (Nitric Oxide Assay Kit, EMSNO, Pierce, Rockford, IL). Briefly, nitrate reductase and the enzyme cofactor were added to wells and incubated to convert nitrate to nitrite. Next, the enhancers were added and incubated at room temperature. Finally, the Griess reagents were added and the samples were analyzed with a microplate reader.

### 2.6 Reactive oxygen species (ROS) level detection

The concentrations of ROS in the culture cells were measured following the manufacturer’s instructions (ROS Assay Kit, S0033S, Beyotime, Shanghai, China). In brief, the ROS Kit was mixed with the ROS inducer. The cells were treated with the mixture then in the positive control group and then incubated for 30–60 min, after which the supernatants were removed and discarded in the detection mix liquid. Finally, cell samples were washed and analyzed with a microplate reader.

### 2.7 NEP activity assay

NEP activity was determined with a Neprilysin Activity Assay Kit (MAK350, Sigma-Aldrich, St. Louis, MO). Briefly, substrate solutions consisting of 1 mM DAGPNG and 10 mM enalaprile in 50 mM Tris-HCl with and without 10 mM phosphoramidon, a NEP inhibitor, were prepared. The samples (50 µg) were incubated at 37°C for 20 min with 100 mL of each substrate solution. The reaction was stopped by boiling for 10 min at 90°C. The samples were then diluted 1:10 with 50 mM Tris-HCl and spun for 5 min in a microfuge at 4°C. The change in fluorescence of the supernatants was monitored by a luminescence spectrometer at an emission wavelength of 562 nm and an excitation wavelength of 342 nm.

### 2.8 Statistical analysis

All experiments were repeated at least three times. The data were listed by mean ± SD. Data were assessed using Student’s *t*-test. A probability value *P* < 0.05 was considered reflective of a statistically significant difference.

## 3 Results

### 3.1 T4 upregulated NEP levels in N2a cells

**Figure 1:**
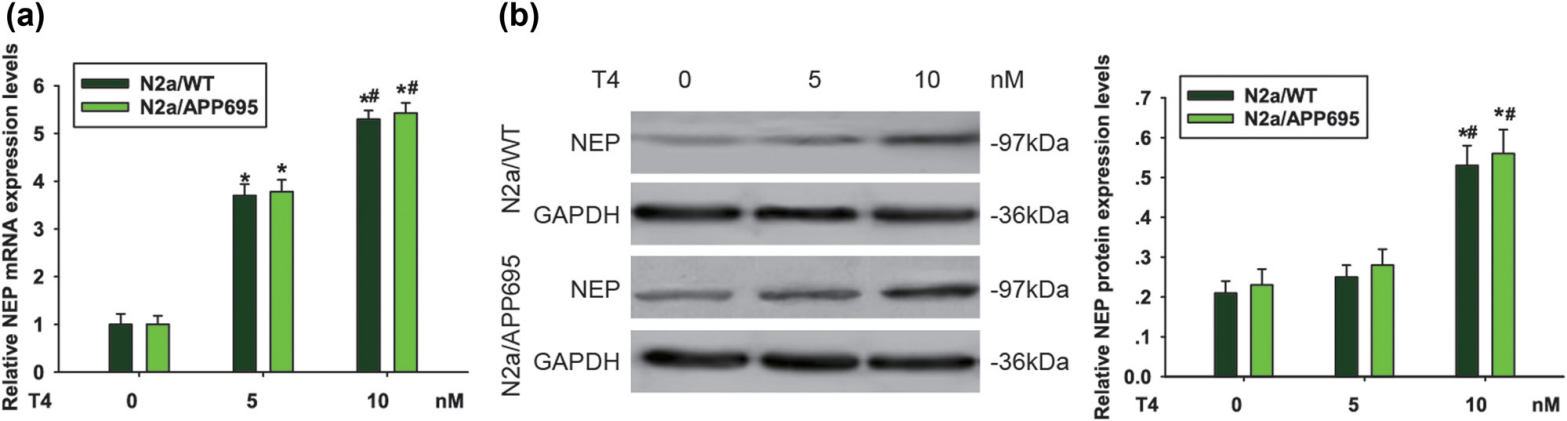
T4 upregulated NEP mRNA and protein levels in N2a cells. N2a/WT and N2a/APP695 cells were treated with 5 nM or 10 nM T4 for 24 h. RT-PCR and western blots were used to determine NEP mRNA expression (a) and NEP protein expression (b). ^*^ *P* < 0.05, compared with untreated cells; ^#^ *P* < 0.05, compared with 5 nM T4-treated cells.

After treating N2a/WT cells and N2a/APP695 cells with 5 or 10 nM T4 for 24 h, qRT-PCR results indicated that NEP mRNA expression was upregulated in both N2a/WT cells and N2a/APP695 cells and that the level was higher when treated with 10 nM T4 compared with 5 nM group (*P* < 0.05, Figure 1a). Next, we detected NEP protein expression by western blot. The increase in the NEP protein level after 10 nM T4 administration for 24 h was similar to that of mRNA levels (*P* < 0.05), but the NEP expression in the 5 nM T4 treatment group had no obvious effect on both N2a/WT cells and N2a/APP695 cells (*P* > 0.05, Figure 1b). These outcomes prompted us to assess the effect of 10 nM tripchlorolide treatment on N2a cells. As a result, we used 10 nM T4 in the following studies.

### 3.2 The NEP inhibitor thiorphan induced cell death in T4-treated N2a/APP695 cells

**Figure 2:**
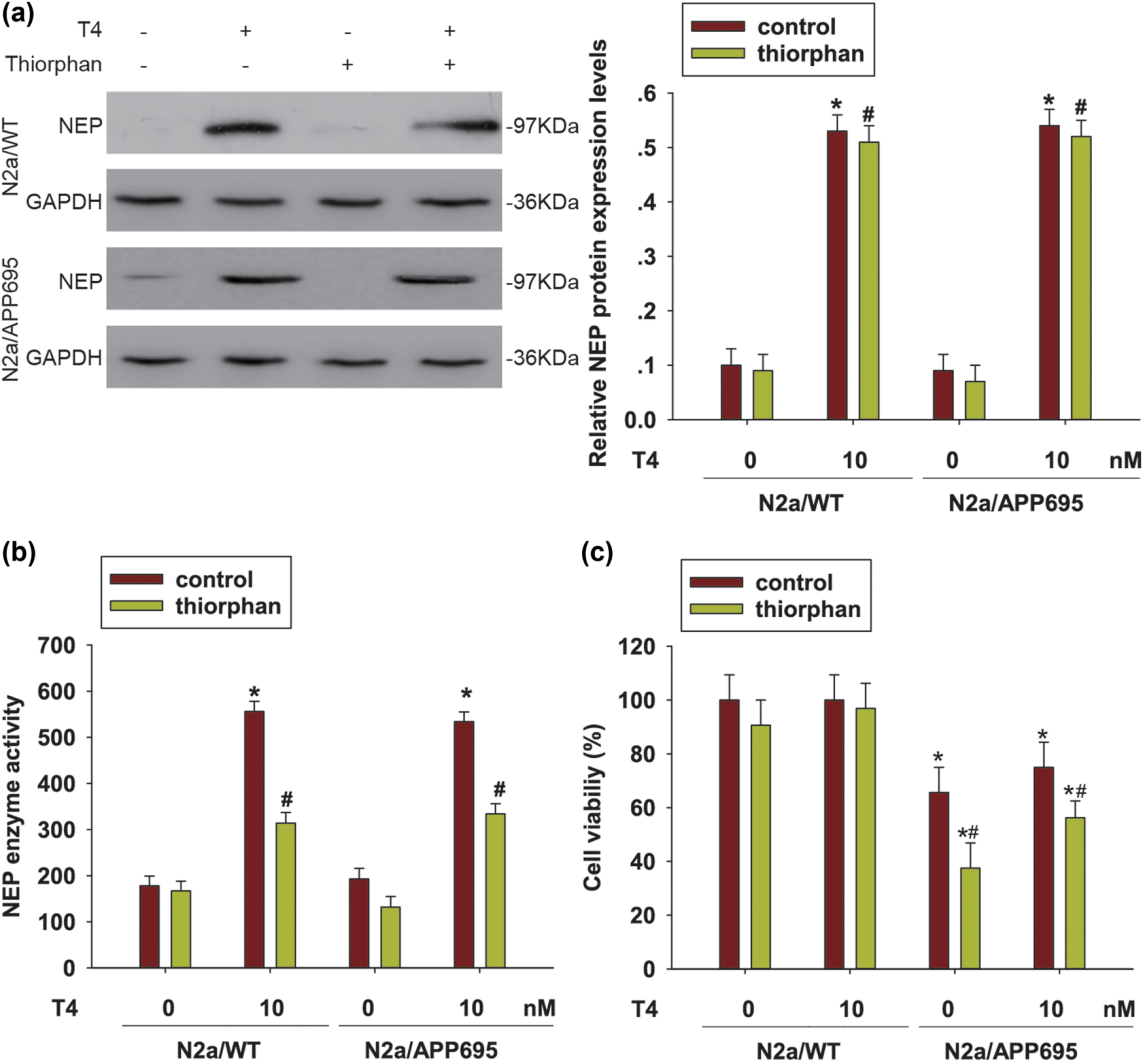
The NEP inhibitor, thiorphan, induced cell death in T4-treated N2a/APP695 cells. (a) The NEP specific inhibitor, thiorphan (500 nM), was used to suppress the NEP enzyme activity in N2a/WT or N2a/APP695 cells following T4 treatment. Western blots were used to detect NEP expression. ^*^ *P* < 0.05, compared with control cells; ^#^ *P* < 0.05, compared with thiorphan-treated cells. (b) NEP activity was detected following the manual protocol. ^*^ *P* < 0.05, compared with T4-untreated control cells; ^#^ *P* < 0.05, compared with T4-treated control cells. (c) MTT assay was performed to assess cell viability. ^*^ *P* < 0.05, compared with N2a/WT cells; ^#^ *P* < 0.05, compared with control group of N2a/APP695 cells.

To investigate the function of NEP activation in neuron injury in AD, a specific NEP inhibitor thiorphan has been used to suppress the NEP enzyme activity. After treating cells with 500 nM thiorphan, NEP expression was found to induce no significant changes in N2a/WT cells or N2a/APP695 cells in both T4-treated or -untreated cells (*P* > 0.05, Figure 2a). When detecting the NEP activity in these cells, we found that thiorphan reduced NEP enzyme activity in T4-treated N2a/WT cells and N2a/APP695 cells (*P* < 0.05, Figure 2b). Next, we investigated cell viability by the MTT assay when treated with T4 pretreated with thiorphan. As shown in Figure 2c, the cell viability was high in N2a/WT cells compared with N2a/APP695 cells (*P* < 0.05). When treated with T4, cell vitality was not affected (*P* > 0.05) but thiorphan reduced the cell survival rate in N2a/APP695 cells when the cells were administered T4 (*P* < 0.05). These data indicated that the effect of T4 might be relevant to NEP.

### 3.3 NEP activation was involved in Aβ secretion in N2a/APP695 cells

**Figure 3:**
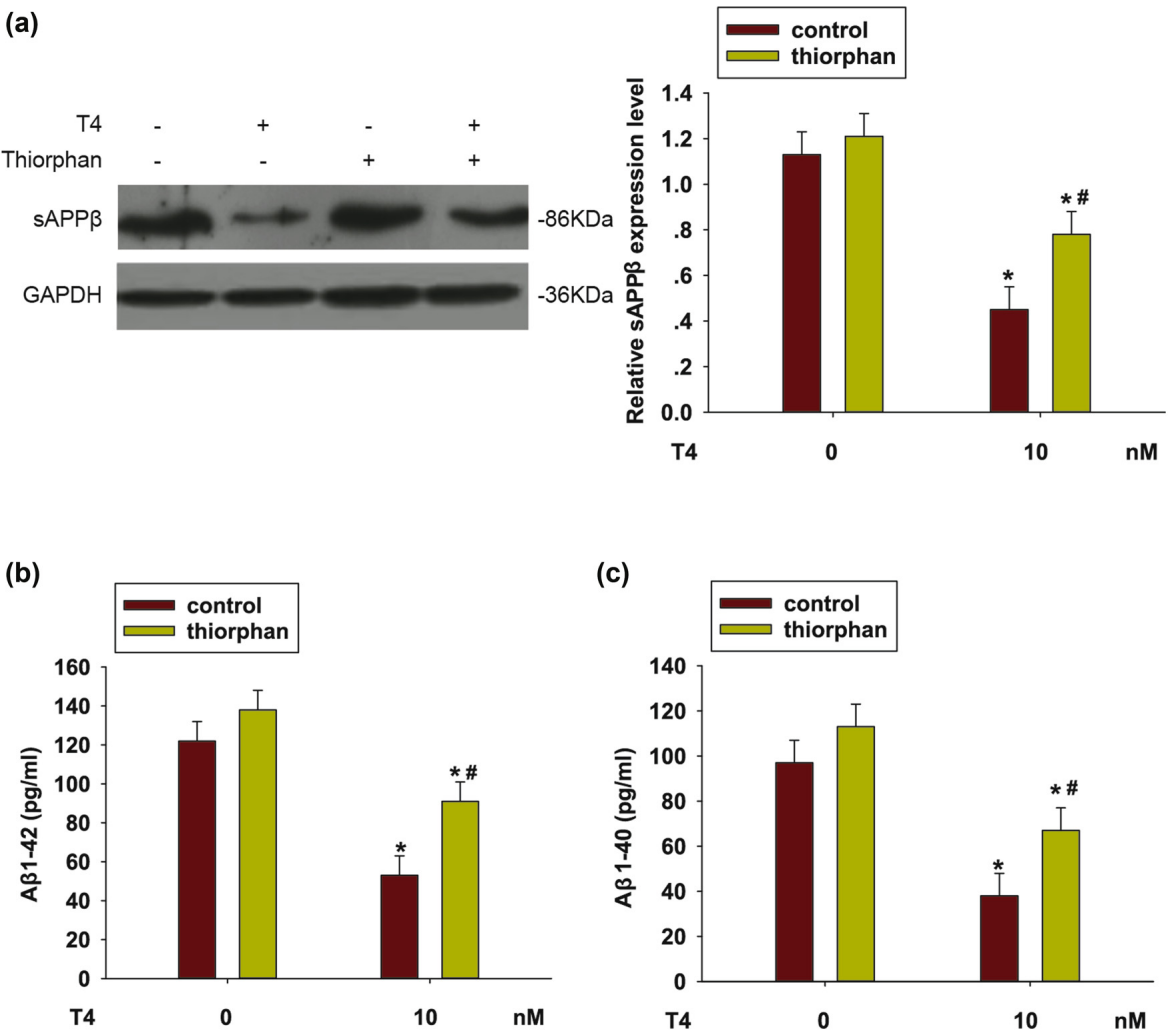
NEP activation involved in T4 reduced Aβ secretion in N2a/APP695 cells. (a) The NEP specific inhibitor, thiorphan (500 nM), was used to suppress the NEP enzyme activity in N2a/WT or N2a/APP695 cells following T4 treatment. Western blotting was used to detect sAPPβ expression. (b) Aβ1-42 and (c) Aβ1-40 levels in the supernatant were also detected by ELISA. *P < 0.05, compared with untreated cells; ^#^ *P* < 0.05, compared with thiorphan-treated cells.

As a major protease, NEP cleaved and degraded β-amyloid. After treatment with T4, the soluble N-terminal cleaved fragment sAPPβ was reduced by the treatment with T4 (*P* < 0.05) but increased when cells were pretreated with thiorphan (*P* < 0.05, Figure 3a). Furthermore, the Aβ1-42 and Aβ1-40 levels in the supernatant were also detected by the ELISA assay. As shown in Figure 3b, the expression levels of Aβ1-42 and Aβ1-40 were decreased when cells were treated with T4 (*P* < 0.05) but the suppression was reversed when cells were pretreated with the NEP inhibitor (*P* > 0.05). These results indicated that NEP might be involved in Aβ cleavage and secretion suppressed by T4.

### 3.4 NEP activation was involved in T4 ameliorated oxidative stress and nitrosative stress in N2a/APP695 cells

**Figure 4:**
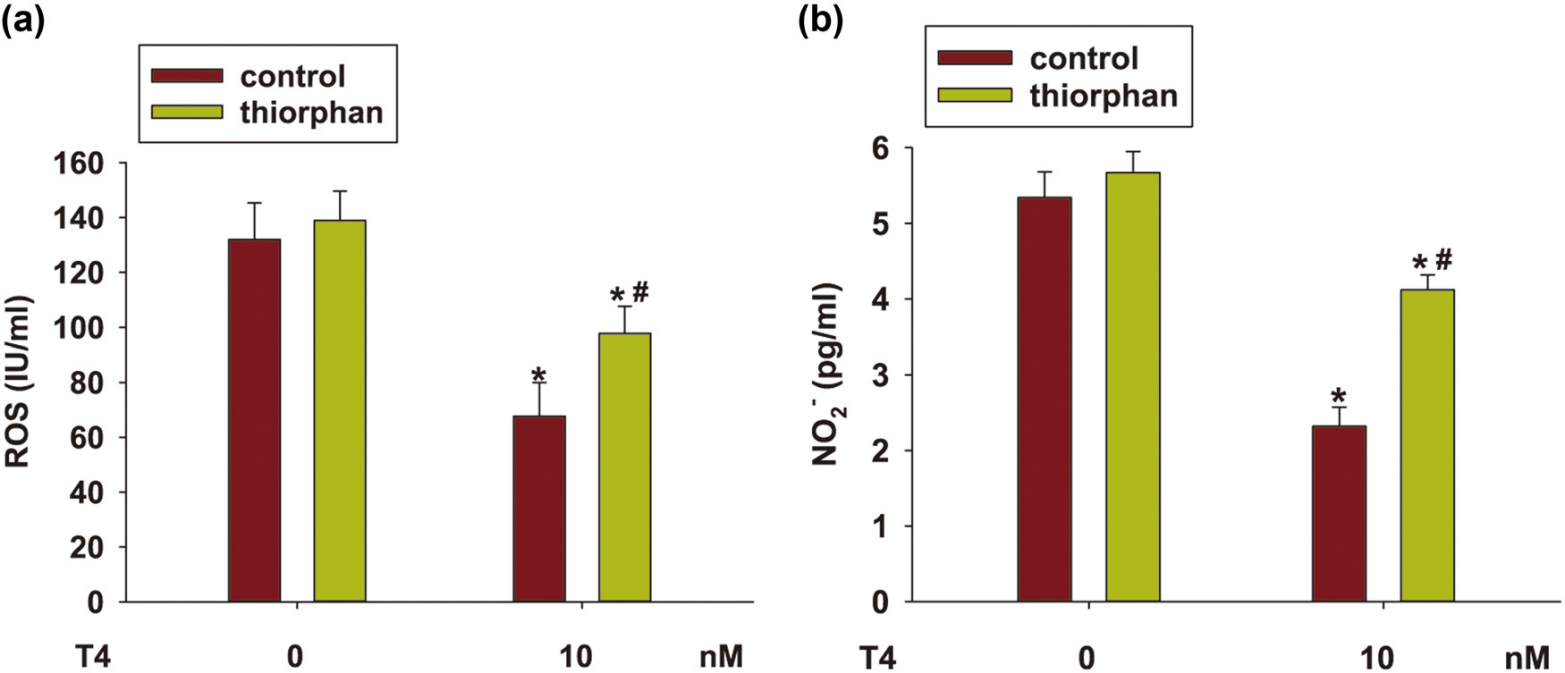
NEP activation involved in T4 reduced ROS and NO production in N2a/APP695 cells. Thiorphan (500 nM) was used to inhibit the NEP enzyme activity in N2a/APP695 cells following T4 treatment. ROS (a) and NO (b) levels in the supernatant were detected. ^*^ *P* < 0.05, compared with untreated cells; ^#^ *P* < 0.05, compared with thiorphan-treated cells.

As oxidative stress and nitrosative stress have been observed in the brain of AD patients, oxidative stress and nitrosative stress were observed in N2a/APP695 cells. T4 pretreatment significantly decreased the levels of ROS and NO (*P* < 0.05, Figure 4). Furthermore, we investigated the changes in oxidative stress and nitrosative stress in cells when treated with T4 pretreated with thiorphan. As shown in Figure 4, the levels of ROS and NO reduced by T4 in the N2a/APP695 cells were reversed with thiorphan treatment (*P* < 0.05). These findings indicated that the neuroprotective effect of T4 was related to its anti‐oxidative and nitrosative stress ability.

### 3.5 NEP activation took part in T4 ameliorated neuroinflammation in N2a/APP695 cells

**Figure 5:**
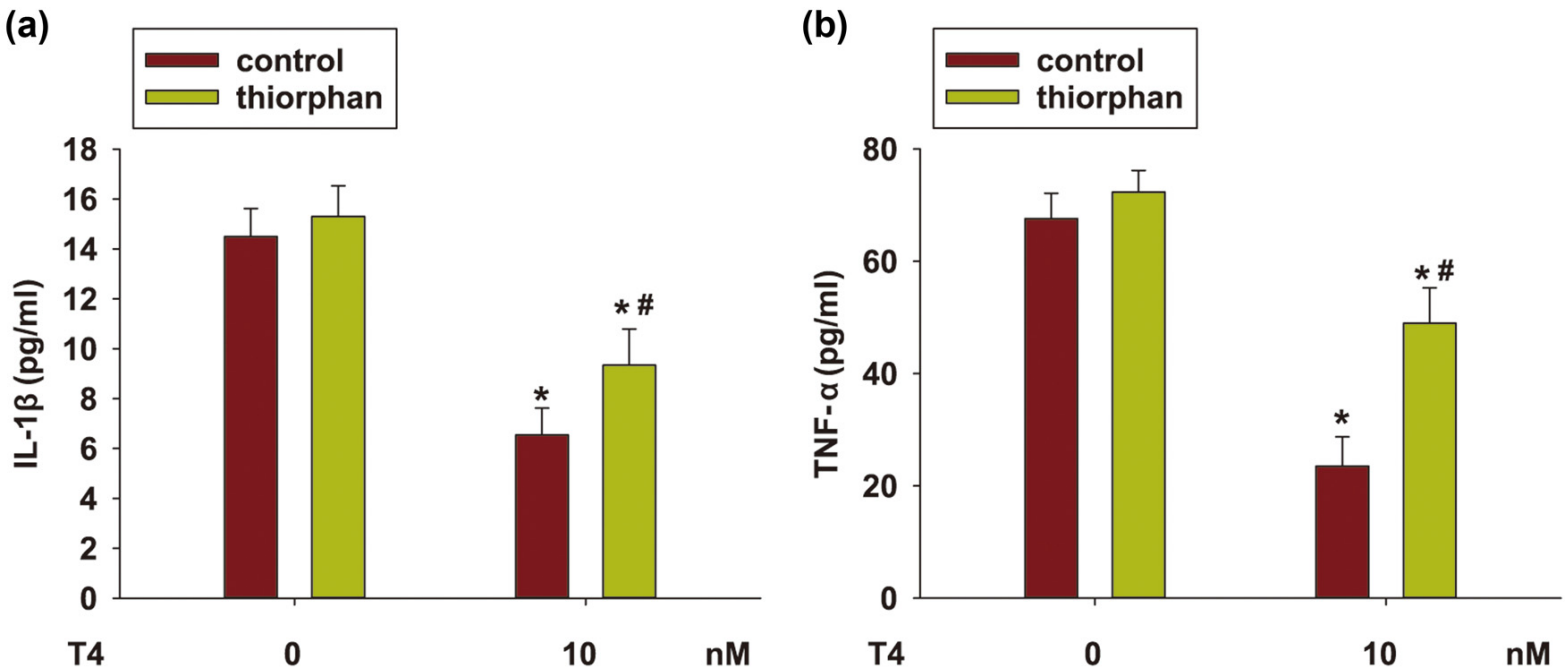
NEP activation involved in T4 reduced neuroinflammation in N2a/APP695 cells. Thiorphan (500 nM) was used to suppress the NEP enzyme activity in N2a/APP695 cells following T4 treatment. Levels of inflammatory factors in the supernatant were accessed by ELISA: (a) level of IL-1β and (b) level of TNF-α. ^*^ *P* < 0.05, compared with untreated cells; ^#^ *P* < 0.05, compared with thiorphan-treated cells.

Aβ accumulation is able to induce neuroinflammation in the brain of AD patients as well. In our research, neuroinflammatory factors, including TNF‐α and IL‐1β, were increased in N2a/APP695 cells. T4 treatment significantly downregulated the expression of TNF‐α and IL‐1β (*P* < 0.05, Figure 5). However, the influence of T4 was reversed when the N2a/APP695 cells were pretreated with thiorphan (*P* < 0.05). These findings suggested that T4 induced NEP activation was related to its anti‐inflammatory ability.

## 4 Discussion

AD is a progressive neurodegenerative disease, with increasing morbidity around the world. In this study, we discovered that T4 induced NEP expression and its medicated Aβ degradation in N2a-APP695 cells, which is always used as the *in vitro* model of Aβ generation. Furthermore, the effect of T4 was suppressed by the NEP inhibitor thiorphan. We identified a new molecular mechanism by which T4 could reduce Aβ production. The results of this study might offer a new theoretical basis for the treatment of AD by T4.

Aβ is known as an original cause of AD, and a number of risk or pathogenic genes of AD contribute to Aβ generation and degradation pathways. In addition, NEP is the major Aβ-degrading protease [16]. Since NEP expression and activity are reported to be decreased in AD, it may be that upregulation of NEP expression or activity may have beneficial effects [17]. On the other hand, oxidative stress, which is enhanced in the brain of AD patients, is associated with the decreased half-life and enzyme activity of NEP [9,18]. In our previous research, we demonstrated that T4 can improve cognitive impairment and promote synaptic plasticity by reducing Aβ levels *in vivo* [15]. In this current study, we found that T4 could promote NEP enzyme activity and reduce the production of Aβ. In addition, T4 was able to reduce oxidative stress and nitrosative stress. These results were similar to previous studies and further proved the role of T4 in the production of AD.

A number of research studies have already indicated that neuroinflammation plays a critical role in the occurrence of AD [19]. Previous studies suggested that the inflammatory response of AD is mainly produced by microglia and astrocytes [20]. In this process, the interaction between glial cells can form an inflammatory waterfall reaction, leading to the amplification of inflammation and the induced neuronal damage [21]. However, recent studies have found that neurons have an inflammatory response, which may be earlier than the glial-activation-induced inflammation [22]. During the development of AD, the accumulated protein Aβ may play a role by inducing signal transduction in neurons [23]. Studies have shown that over the course of this process, Aβ protein can activate toll-like receptors, which in turn promote NF-κB signal transduction, thereby promoting neurons to produce inflammatory factors [24]. In addition, in the early stage of AD, the release of inflammatory factors in neurons is considered a pivotal link in the progression of AD [25]. The inflammatory factors released at this time can promote the activation of astrocytes and microglia, leading to the generation of inflammatory responses in the brain [26]. In our research, we found that T4 could inhibit the release of inflammatory factors of neurons containing TNF‐α and IL‐1β by regulating NEP enzyme activity. This result suggested that the role of T4 in protecting neurons may be linked to the early inhibition of the production of Aβ to inhibit the neuroinflammatory reaction, thereby delaying the pathogenesis of AD.

However, there remain some limitations to our experiments. For instance, the effects of T4 on NEP *in vivo* warrants further study. On the other hand, whether T4 directly or indirectly interacts with NEP to enhance its activity still remains unclear. Due to the limitations of the experimental conditions, we will focus on the mechanism of T4 interaction with NEP in future work.

In conclusion, we found that T4 induced NEP expression and its medicated Aβ degradation, as well as secretion, in N2a-APP695 cells, which might be a novel therapy target paving the way for drug development. Our research indicated that the administration of T4 may be a potential therapy for AD.
